# Genome Sequence of a Clinical Strain of *Acinetobacter baumannii* Belonging to the ST79/PFGE-HUI-1 Clone Lacking the AdeABC (Resistance-Nodulation-Cell Division-Type) Efflux Pump

**DOI:** 10.1128/genomeA.00962-16

**Published:** 2016-09-08

**Authors:** M. López, L. Álvarez-Fraga, E. Gato, L. Blasco, M. Poza, L. Fernández-García, G. Bou, M. Tomás

**Affiliations:** a Department of Microbiology, Complejo Hospitalario Universitario A Coruña-INIBIC, La Coruña, Spain; b Spanish Network for the Research in Infectious Diseases (REIPI RD12/0015), Hospital Universitario Virgen Macarena, Sevilla, Spain

## Abstract

Increased expression of chromosomal genes for resistance-nodulation-cell division-type efflux systems plays a major role in the multidrug resistance of *Acinetobacter baumannii*. Little is known about the genetic characteristics of clinical strains of *Acinetobacter baumannii* lacking the AdeABC pump. In this study, we sequenced the genome of clinical strain Ab421 GEIH-2010 (belonging to clone ST79/PFGE-HUI-1 from the GEIH-REIPI Ab. 2010 project) which lacks this efflux pump.

## GENOME ANNOUNCEMENT

*Acinetobacter baumannii* is an important pathogen that causes nosocomial infections associated with high morbidity and mortality (1). Three resistance-nodulation-cell division-type efflux pumps have been described in the *A. baumannii* clinical strains AdeABC, AdeIJK, and AdeFGH. The AdeABC efflux pump confers high-level resistance to several antibiotics and is regulated by the two-component AdeRS system (2, 3). A type of TetR-type transcriptional regulator (AdeN) has been associated with overexpression of the AdeIJK efflux pump involved in resistance to several antimicrobials (4). Finally, the AdeFGH pump also confers high-level resistance to multiple antimicrobials (4). Overexpression of this pump has been associated with a mutation of a LysR-type transcriptional regulator (LTTR), named AdeL and located upstream from the *adeFGH* operon and transcribed in the opposite direction (5).

In this study, we report a draft genome sequence of Ab421 GEIH-2010 clinical strain (belonging to clone ST79/PFGE-HUI-1). Genomic DNA was isolated using the Wizard genomic DNA purification kit (Promega) following the manufacturer’s protocols. Genome sequencing was performed using the GS Junior sequencer (454 Life Sequencing Inc., Branford, CT, USA). Genomic shotgun libraries were prepared from 1 µg of DNA and processed according to the manufacturer’s instructions to load each sample on half of a picotiter plate. Standard flowgram format (SFF) files were generated after images had been processed by the shotgun pipeline. Only nucleotides labeled in the SFF file as high quality were extracted in a FastQ file. Final sequences were obtained by removing sequencing adaptors and 3′ low-quality ends. The consensus sequence was obtained using SAMTools (6) and aligned with the *A. baumannii* ATCC 17978 bacterial chromosome using the BLAST2seq tool (7). The consensus sequence is also provided in a FASTA file (cns.fasta). Variant identification was performed by sequence comparison against the reference genome, implemented using the Genome Analysis Toolkit (GATK) (8) with default parameters. The genome was functionally annotated using the RAST server (9).

The genome, which includes a chromosome of 3,976,764 bp with a G+C content of 39.92%, showed low homology to other *Acinetobacter* genomes included in the RAST server. The closest neighbor was *A. baumannii* TYTH-1 (score 529). The scores for *A. baumannii* ATCC 17978 and *A. baumannii* AB307-0294 were 445 and 343, respectively. A total of 3,066 coding regions were found in the genome, of which 2,189 (71%) were able to be functionally annotated. These coding regions belong to 392 different subsystems. The number of RNAs was 86. The annotated genome has 47 genes responsible for antibiotic resistance and toxic compounds, including 14 genes coding for the efflux pumps AdeIJK and AdeFGH associated with the multidrug resistance profile. Interestingly, AdeN, which was located 813 kb upstream from the AdeIJK efflux pump, did not have mutations associated with overexpression of the AdeIJK pump. However, the *Acinetobacter* genome had a LysR-type regulator protein (AdeL) upstream of the AdeF protein. Surprisingly, translation of this protein was interrupted in the Ab421 GEIH-2010 genome due to the appearance of a new mutation that introduced an amino acid substitution (Met7→Stop).

### Accession number(s).

The genome sequence of Ab421 GEIH-2010 strain has been deposited at GenBank under the accession number CP014266. This genome sequence is part of a II Spanish multicenter study, GEIH-REIPI Acinetobacter baumannii 2000-2010 project (PRJNA308422).
